# mltG gene deletion mitigated virulence potential of *Streptococcus mutans*: An *in-vitro, ex-situ* and* in-vivo* study

**DOI:** 10.1186/s13568-023-01526-x

**Published:** 2023-02-20

**Authors:** Sahar Zaidi, Khursheed Ali, Yadya M. Chawla, Asad U. Khan

**Affiliations:** 1 Medical Microbiology and Molecular Biology Laboratory, Interdisciplinary, Biotechnology Unit, Aligarh Muslim University, Aligarh, 202002 UP India; 2 ICGEB-Emory Vaccine Center, International Centre for Genetic Engineering and Biotechnology, New Delhi, India

**Keywords:** *S. mutans*, mltG, Virulence, Biofilm, Proteins, Dental caries

## Abstract

Bacterial cells are surrounded by a peptidoglycan (PG) cell wall, which is essential for cell integrity and intrinsic biogenesis pathways; hence, the cell wall is a potential target for several antibiotics. Among several lytic transglycosylases (LTs), the mltG gene plays a crucial role in the synthesis of peripheral PG. It localises the re-modelled PGs for septum formation and cleavage across the bacterial cell wall during daughter cells separation. However, the role of mltG gene in bacterial virulence, particularly in Gram-positive bacteria during dentine biofilm and caries development, has remained unexplored. Hence, we exploited Gram-positive *Streptococcus mutans* cells for the very first time to construct a mltG knock-out bacterial strain, e.g., ΔmltG *S. mutans*. Systematic comparative investigations revealed that doubling time (Td), survival, enzymatic efficiencies, pH tolerance, bio-synthesise of lipid, proteins and DNA, biofilm formation and dentine lesions were significantly (p < 0.001) compromised in case of ΔmltG *S. mutans* than wild type strain. The qRT-PCR based gene expression profiling revealed that transcriptional expression of critically important genes involved in biofilm, metabolism, and stress response were dysregulated in the mutant. Besides, an incredible reduction in dentine caries development was found in the molar teeth of Wistar rats and also in human extracted teeth. Concisely, these trends obtained evidently advocated the fact that the deletion of mltG gene can be a potential target to impair the *S. mutans* virulence through severe growth retardation, thereby reducing the virulence potential of *S. mutans*.

**Graphical Abstract:**

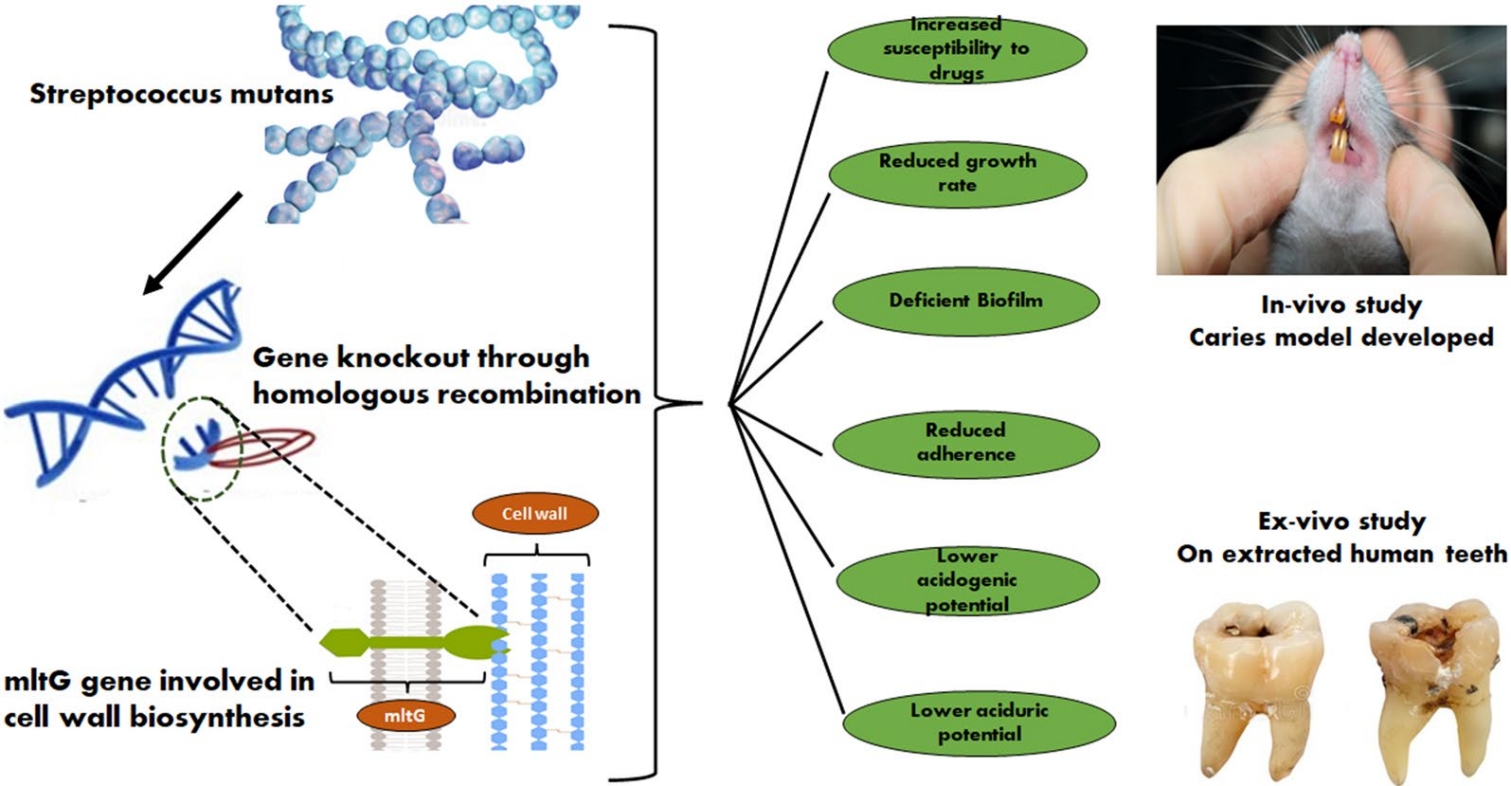

**Supplementary Information:**

The online version contains supplementary material available at 10.1186/s13568-023-01526-x.

## Key Points

- *S. mutans* is regarded as the most notorious cariogenic pathogen in human oral microbiome.
- mltG gene acts as terminase and endolytically controls the PG strand elongation in bacteria.
- Compromised virulence potential of ΔmltG w.r.t. wild type also validated through in-vivo study.

**Supplementary Information:**

The online version contains supplementary material available at 10.1186/s13568-023-01526-x.

## Introduction

Amongst tooth health concerns, the formation of dental caries (DCs) is the most prevalent chronic oral disease in both, children and adults of our modern society (Miglani 2019). Shockingly, ~ 90% of school going children and ~ 100% of old age humans survive with the pain and discomfort of dental caries in low socioeconomic status groups worldwide (Petersen et al. 2005; Schwendicke et al. 2015; Fejerskov and Kidd 2008). DC is a multifactorial, chronic illness caused by oral microbiota under non-ideal conditions in the oral cavity. The genesis of DC encompasses the gradual demineralization of hydroxyapatite of tooth surface through metabolic products, e.g., organic acids. Organic acids are yielded by cariogenic consortia resident bacteria present on tooth surfaces adhered biofilms by utilising available sugars (Fejerskov and Kidd 2008; Loesche 1986). Surprisingly, a single Gram-positive pathogenic *Streptococcal* species, among 49 other species in human oral microbiome, can act as a potential infectious agent to lead infections in diverse anatomic spaces, including skin, soft tissue, endocarditis, pneumonia, meningitis, sinusitis, otitis media, chorioamnionitis, sepsis, and even death, in different age cohorts (Guevara et al. 2020; Kasper et al., 2021). Owing to its excellent intrinsic ability to produce sticky glucans, *S. mutans* is often regarded as the most notorious cariogenic pathogen in the human oral microbiome (Birlutiu et al. 2018; Hamada and Slade 1980). Such glucans with several surface encapsulating auxiliary proteins surround the *S. mutans* and thereby facilitate their colonisation in biofilm on the tooth surface. Additionally, its ability to generate and tolerate organic acids plays a crucial role in the virulence of *S. mutans*. In fact, glycolytic lactic acid production triggers the pH drop and creates an acidic salivary environment, which accelerates the demineralization of teeth and promotes caries formation. Thus, to withstand such an adverse acidic environment being created on the human tooth surface, *S. mutans* elicits certain transcriptional and physiologic modulations to overcome the acid-damage to its DNA synthesis and metabolic machinery that is collectively termed the "acid tolerance response (ATR) (Banas and Vickerman 2003; Sztajer et al. 2014; Ahn et al. 2006; Lemos et al. 2005; Guo et al. 2015).

Notably, PG building blocks construct tough yet flexible protective cell wall architecture which is prone to have subtle abortive event either during bio-genesis or remodelling of PGs and can consequent in deleterious kinks and pits in cell wall. In fact, during bacterial cell division, NAG-NAM (i.e., NAG: N-acetylglucosamine and NAM: N-acetylmuramic acid) polysaccharide units (n) fuel the biosynthesis of PGs through two distinct sets of enzymes: (i) polymerases and (ii) lytic transglycosylases (LTs), recruited to connect and re-model the PG building blocks, respectively, to accommodate the next PG unit and associated protein assemblies. Each PG strand remains attached covalently to its neighbouring strand via peptide stems linked with NAM saccharide, resulting in a cross-linked, net-like PG layer. This whole polymer is known as the sacculus that encapsulates the bacterial cell and maintains the cell shape (Jorgenson et al. 2014; Winther et al. 2021; Sassine et al. 2021; Bohrhunter et al. 2021; Yunck et al. 2016). However, a certain level of hydrolysis in septal PGs is essential in order to (i) create space for incorporation of newly generated PG building-blocks and (ii) facilitate the separation of two daughter cells during cell division, mediated by dynamic and transient multi-protein complexes, the elongasome and divisome (Wientjes et al. 1991; Silhavy et al. 2010; Vollmer and Seligman 2010; Egan et al. 2020). For this, LTs congregate with other proteins as well as enzymes to form machinery that crumbles this layer of PG to release its constituent polysaccharides such as NAG and NAM (Williams et al. 2020; Walter and Meyer 2019; Viala et al. 2004). This conversion is the hallmark of LT catalysis. Beyond cell wall regulation, the products generated from LTs catalysed reactions can induce potential defence mechanisms in bacterial cells, such as β-lactam drugs resistance mechanisms in Gram-negative *P. aeruginosa* and several *Enterobacteriaceae*. The β-lactams are targeted by penicillin-binding proteins (PBPs) to delay cell-wall synthesis (Lee et al. 2003, 2001; Pratt 2016). This imitation consequently deactivates PBPs and causes ineffective crosslinking of the PG. Later, such cross-linked PGs are degraded by LTs. Nevertheless, enabling the role of LTs intimately relates muropeptide recycling to antibiotic resistance. Despite their critical role in bacterial physiology and resistance against antibiotics, LTs remain unexplored to be considered as significant drug targets. Besides, LTs can also induce cytotoxicity and NF-B-dependent adverse innate immune responses in the host (Knilans et al. 2017). Therefore, in the present study, we engineered the genome of the *S. mutans* strain by knocking out the mltG gene (one of the lytic transglycosylases) to explore whether it is involved in the bacterial virulence by using ex-situ human and *in-vivo* animal tooth models.

Obtained results exhibited a significant decrease in different virulence attributes in the mltG deficient construct (ΔmltG) as compared to its wild-type counterpart in *in-vitro*, *ex-vivo*, and in the oral cavity of experimental Wistar rats. Our results and an in-depth review of published literature prompted us to conceive a hypothesis, that can address how the mltG gene governs the activities of many proteins and enzymes. The results obtained in the present study are fascinating and provocative for the researchers who are exploring strategies to control the pathogenicity of different bacteria by attenuating a particular protein or enzyme present in the complex milieu of indigenous biomolecules. To our best knowledge, such comparative and systematic evaluation of mltG induced virulence paradigm has never been studied in Gram-positive cariogenic human pathogen *S. mutans* and there are only a few studies which have exhibited its role only in cell wall remodelling and PG metabolism (Winther et al. 2021; Sassine et al. 2021; Bohrhunter et al. 2021; Yunck et al. 2016).

## Materials and methods

### Declarations

This study was carried out following all the institutional ethical standards. The research on rats was conducted with the approval of "Jawaharlal Nehru Medical College, AMU, Institutional Animal Ethics Committee," registration no. 401/GO/Re/S/2001/CPCSEA. Besides research on extracted human teeth was performed with the approval of “Dr. Ziauddin Ahmad Dental College AMU”, “AMU ethical committee”, registration no {(No. 151/201517/PDFWM-2015-2017-UTT-31140 (SAII)}. Each applicable guideline, whether international, national, and/or institutional, has been followed for the use of animals.

### Bacterial stains and culture conditions

*S. mutans* MTCC497 was procured from the Institute of Microbial Technology, Chandigarh, India. Cells in each experiment were grown in Todd-Hewitt (TH) broth supplemented with 0.3% yeast extract and 1% sucrose (Himedia Labs, Mumbai, India) at 37 °C under aerobic conditions. However, Mitis salivarius (MS) broth containing appropriate antibiotic was employed for the strain selection during the *in-vivo* study. Kanamycin (500 µg/mL) was added to the TH medium, according to the desired experimental conditions for mutant strain selection. Plasmid pet28a was employed to amplify the kanamycin cassette. Horse serum was used at 10% of the total volume of media for transformation. Competence was induced by adding a synthetic competence stimulating peptide (CSP) with a purity of > 95% (the amino acid sequence of the peptide is NH2-SGSLSTFFRLFNRSFTQALGK-COOH) to the culture. CSP was purchased from "S" Bio Chem, Kerela, India. A 1 mg/mL stock of synthetic CSP was prepared by dissolving the peptide in Milli Q water, stored at − 20 °C. Table 1 lists the primers used in qRT- PCR. All the experiments that have been performed in this study were replicated three times biologically.

**Table 1:** Determination of MIC (minimum inhibitory concentration) and MBC (minimum bactericidal concentration) of three different antibiotics against wild type and ΔmltG type *S. mutans*

Antibiotics	Mode of action	Group of antibiotic	MIC (µg/mL)	
			Wild type* S. mutans*	ΔmltG *S. mutans*
Vancomycin	Inhibits cell-wall synthesis	Glycopeptide	312.5	78.0
Gentamycin	Inhibits protein synthesis	Aminoglycoside	312.5	19.0
Ciprofloxacin	It inhibits DNA replication	Fluoroquinolones	625.0	312.5

### Construction of the mutant by mltG gene deletion

**Fig. 1:**
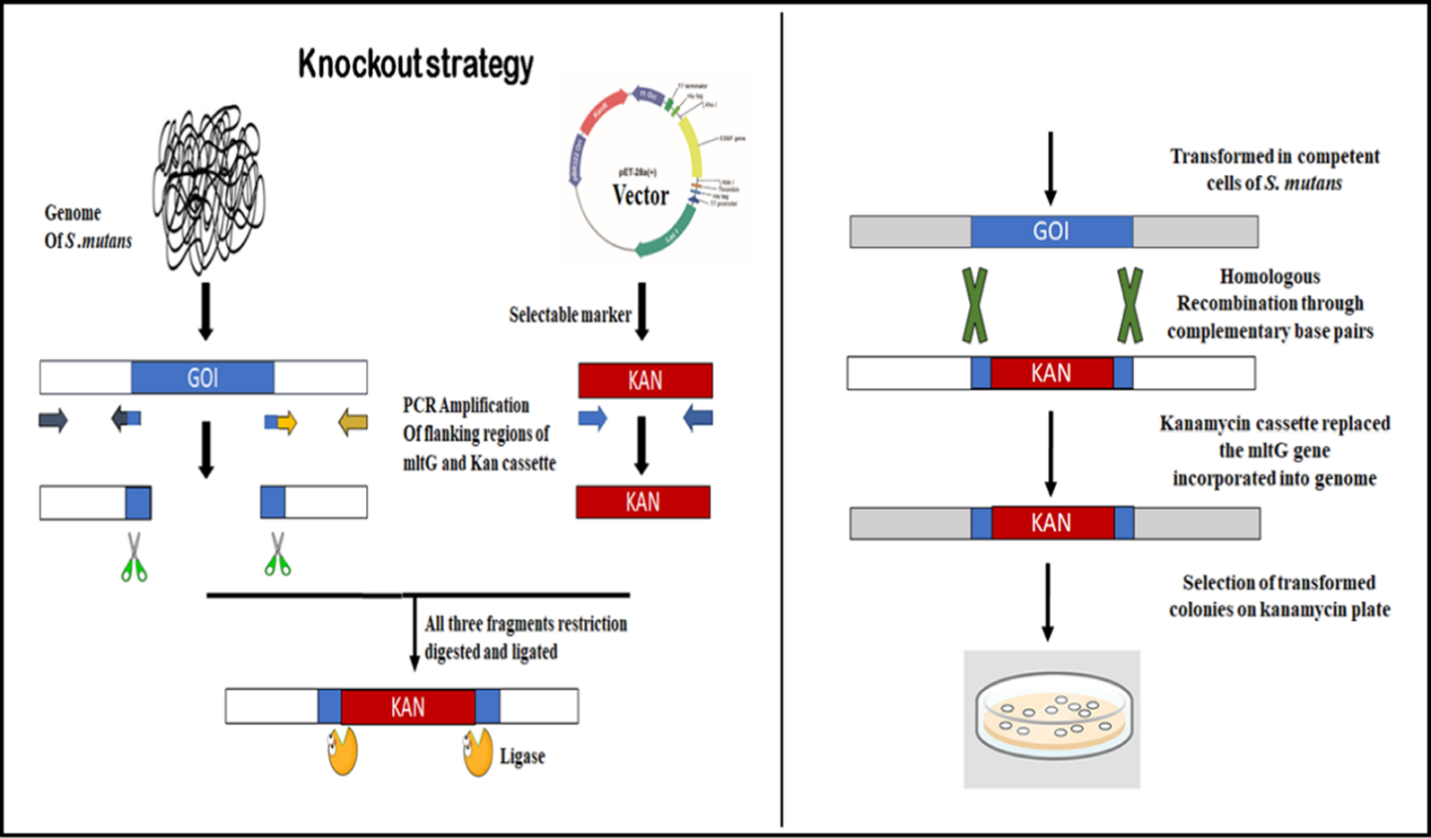
Illustrates the knockout strategy adopted in the study to delete the mltG gene where, GOI = gene of interest, Kan = kanamycin cassette

Figure 1 demonstrates the scheme we adapted to construct ΔmltG in the *S. mutans* strain (MTCC 497). Indeed, the mutant was constructed by following an insertion-deletion strategy. The primers involved in knockout and to confirm gene deletion were enlisted in Table 1. Concisely, mltG-up (927 bp) and mltG-dw (983 bp) fragments containing upstream and downstream regions along with some sequences of the mltG gene were amplified with mltG-upF and mltG-upR and mltG-dwF and mltG-dwR primers, respectively. Whereas, PcKan (815 bps) from plasmid pet28a was amplified using KanF and KanR primers (Table 1). Next, the amplicons underwent restriction digestion with Pst1 and Bam H1 and were successively ligated to create a mltG-up::PcKan::mltG-dw fragment. Finally, this fragment was transformed into *S. mutans* with the aid of synthetic CSP. Through double-crossover homologous recombination, the PcKan replaced the internal region of the mltG. Template genomic DNA was extracted from cultures grown on TH agar plates, as described elsewhere (Senadheera et al. 2005; Zeng et al. 2006). Incorporation of the Kan marker at the appropriate locus was validated through PCR and sequencing (data provided in the Additional file 1: Figs. S1 and S2). Separate primer pair for the mltG gene, i.e., mltGF and mltGR, was used with the correctly predicted size from the mutants (1074 bp), which contain a kanamycin cassette flanked by some base pairs of the mltG region in the mltG deficient strain. Wild-type MTCC 497 chromosomal DNA was used as a negative control.

### Influence of mltG deletion on MICs of antimicrobial agents against *S. mutans*

The broth microdilution method was employed to assess the MICs of the antibiotics acting on different targets as defined earlier (Zaidi et al. 2020). Two-fold microdilutions of antibiotics, viz., gentamycin, vancomycin, and ciprofloxacin, were made in THB (100 µL/well) in 96-well microtiter plates, separately. The designated wells were seeded with wild and ΔmltG *S. mutans* cells (~ 107 CFU/mL) for each of the antibiotics separately and kept at 37 °C for 48 h.

### Influence of mltG deletion on the growth dynamics of *S. mutans*

Overnight grown wild and ΔmltG *S. mutans* cells were added to 50 mL fresh THB and allowed to reach the stationary phase separately. The OD600nm of 1 ml aliquots was recorded at an interval of 1 h, up to 20 h under identical conditions, though both the cultures reached the stationary phase after 16 h (Wen et al. 2015; Miller et al. 2015; Padfield et al. 2020).

### Influence of mltG gene deletion on biofilm formation

Visualization of biofilm formed by wild and ΔmltG *S. mutans* strains was carried-out using crystal violet assay (CV), as described elsewhere (Loo et al. 2000). Briefly, ~ 107 CFU/mL of test strains in THB (200 µl) were added to designated wells of a microtiter plate for 48 h at 37 °C. Next, media containing free cells were decanted and wells were dried. The dried biofilms in the wells were stained with CV (0.1%). Subsequently, the CV was removed, wells were dried, and 200µL of ethanol was added into each well. Finally, the absorbance of CV was recorded at OD630nm on a 96-well microtiter plate reader (iMark Microplate Reader, Bio-Rad, 1681130, USA).

### Adherence potential of wild and ΔmltG types of *S. mutans* on glass surfaces

Cultures (0.2 mL) of wild and ΔmltG *S. mutans* were added to 1.8 mL of media containing 1% sucrose in inclined (30°) tubes and kept at 37 °C for 18 h. Broths containing the non-adhered cells were collected in separate tubes, and adhered cells were washed cautiously with 0.5 mL of PBS to remove the loosely bound cells. The broths, containing un-adhered cells were decanted, and the PBS washes were collected, centrifuged, and re-suspended in PBS. The bacterial cells adhered to the glass surface were extracted by adding NaOH (0.5 M), centrifuged, and re-suspended in the same volume of PBS. The bacterial cell density in either of the suspensions was determined by evaluating their turbidity at 600 nm. Total bacterial growth was calculated by summing up the values of turbidity for both adhered and non-adhered bacterial cells. The adherence percentage of wild and ΔmltG *S. mutans* was enumerated as (Segal et al. 1985).

$${\mathbf{Adherance}}\% = \frac{{{\mathbf{Turbidity}}\,{\mathbf{of}}\,{\mathbf{adhered}}\,{\mathbf{bacteria}}}}{{{\mathbf{Turbidity}}\,{\mathbf{of}}\,{\mathbf{total}}\,{\mathbf{bacteria}}}} \times {100}$$

### Biofilm architecture analysis under scanning electron microscopy

Scanning electron microscopy (SEM) was employed to investigate the disruptions in the biofilm formed by ΔmltG *S. mutans*. Briefly, 48 h aged wild and ΔmltG biofilms on glass-coverslips were fixed with 2.5% glutaraldehyde and 2% formaldehyde in PBS for 2 h at 4 °C, followed by serial dehydration with a series of ethanol (20, 40, 60, 80, and 100%). The dried coverslips were then coated with gold and visualised under SEM, as described elsewhere (Misba et al. 2017).

### Biofilm architecture analysis under scanning electron microscopy

Scanning electron microscopy (SEM) was employed to investigate the disruptions in the biofilm formed by ΔmltG *S. mutans*. Briefly, 48 h aged wild and ΔmltG biofilms on glass-coverslips were fixed with 2.5% glutaraldehyde and 2% formaldehyde in PBS for 2 h at 4 °C, followed by serial dehydration with a series of ethanol (20, 40, 60, 80, and 100%). The dried coverslips were then coated with gold and visualised under SEM, as described elsewhere (Misba et al. 2017).

### Acidogenicity evaluation of wild and ΔmltG *S. mutans*

The intrinsic ability of wild and ΔmltG *S. mutans* to produce acid as a metabolic by-product of glycolysis was compared by measuring the pH of cell suspensions, as described elsewhere (Valdez et al. 2017; Gregoire et al. 2007). Briefly, freshly overnight grown wild and ΔmltG *S. mutans* cells were pelleted (1000 rpm, 5 min), washed (PBS) and re-suspended in 200 mL of KCl (50 mM) and MgCl2 (1 mM) mixture, enriched with glucose (55.6 mM) at pH 7.2 maintaining the cell-count (~ 107 CFU/mL). A gradual, time-dependent decline in pH up to a cut-off pH of 3.0 was recorded at different points of time (5–360 min) by using a glass pH electrode and expressed as the area under the curve (AUC).

### Aciduricity assay

Similarly, the ability to withstand acidic stress was assessed by an acid killing assay, as described by Valdez et al. 2017. Precisely, an aliquot (~ 107 CFU/mL) of 18 h aged wild and ΔmltG *S. mutans* cells were added to media containing 1% sucrose and allowed to grow to log phase (OD600nm = 0.5). Next, the cells were pelleted (1000 rpm, 5 min) and washed in PBS vigorously. The pellets were re-suspended in glycine (0.1 M) buffers (200 mL) maintained at different pH values (2.8, 5.0, and 7.0) separately. The acid killing of wild and ΔmltG *S. mutans* cells was evaluated by spreading the 0.1 mL aliquot from all experiments on THB plates after incubating for zero (T0) and 60 min (T60) for each pH value at 37 oC and incubating the plates for 48 h.

### Influence of ΔmltG deletion over virulence genes expressions of *S. mutans* by qRT-PCR analysis

The influence of mltG existence over the transcriptional expressions of co-existing virulence genes, viz. fruA, gtfC, clpA, spaP, vicA, atpA, ropA, comcD, ccpA, ftsA, murE, murN and gbpB, in ΔmltG *S. mutans* were assessed by qRT-PCR analysis. Briefly, freshly grown wild and ΔmltG *S. mutans* cells were taken to extract as-well-as purify RNA by using Tri-reagent (Sigma-Aldrich, St-Louis, USA) extraction protocol, as described by Zaidi et al. 2020. RNA concentrations were estimated by considering valid absorption ratios of A260nm/A280nm (UV–Vis, Shimadzu, USA), whereas the integrity of the RNAs was estimated by performing agarose-gel-electrophoresis. To prepare the transcription (RT) reaction mixture, a high-capacity cDNA RT kit (Applied Biosystems, USA) was employed. 1 μg extracted RNA was taken to make cDNA, which was later stored at − 20 °C. Lastly, using SYBR green master mix, quantitative qRT-PCR was conducted by Step-One software (Applied Biosystems). The reaction mixture for qRT-PCR contained primers and cDNA at a concentration of 100 ng. For primer design, the genome sequence AE014133.2 was used (Table 1). The qRT-PCR cycles include denaturation (10 min, 95 °C, n = 1) amplification (n = 40 with 15 s at 95 °C of denaturation), annealing (30 s, 60 °C) with an extension for 30 s at 72 °C were performed. 16S rRNA was used to normalise the levels of expression of test genes (Misba et al. 2017; Forssten et al. 2010).

### Flow cytometry-based comparative analysis of mltG influence over lipid, protein, and DNA synthesis in exponentially growing planktonic and biofilm-embedded *S. mutans* cells

Flow cytometry (FCM) analysis was carried out to estimate and compare the components of elongasome and divisome of the wild and ΔmltG *S. mutans* cells. Both the strains were allowed to grow exponentially (O.D. 600 nm = 0.5: log phase). Parallel, 48-h-old wild and ΔmltG *S. mutans* cells embedded in biofilm matrices (elongasome and divisome are nonfunctional) were harvested by modest sonication (40 amplitude, 10 pulses of 1 s, Sonics & Materials Inc., USA), centrifugation (5000 rpm, 5 min), and repeated washing in PBS. The obtained pellets were stained with fluorescent dyes; (i) fluorescein isothiocyanate (FITC, 1 µg mL-l, 8 h, 4 oC), (ii) Nile-red, (10 µg/mL, 30 min 37◦C) and cocktail of mithramycin (100 µg/mL, 10 min, 37 oC) and ethidium-bromide (EtBr, 50 µg/mL, 10 min 37 oC), finally diluted to OD600nm = 0.5 in FCM sheath fluid to quantify the proteins, lipid, and DNA contents, respectively. The BD X-20 LSR Fortessa FCM equipped with Flowjo10 data analysing software was employed in these studies. The FCM was first calibrated to detect 10,000 cells (events) per sample. Forward scatter (FSC) and side scatter (SSC) were detected using linear amplification. A detailed FCM acquisition method for each sample has been appended in the supporting information.

### Comparative *in-vivo* dental biofilm and caries Wistar rat model

Caries-susceptible Wistar rats aged 12–14 weeks were used to investigate the *in-vivo* effects of the mltG deletion on dental plaque and fissure caries. Two groups (N = 5 per group) of adult Wistar rats were assigned to be infected with wild and ΔmltG *S. mutans*, separately, whereas, third group was kept uninfected. Initially, to eliminate oral bacteria in teeth, all groups were treated with cotton swabs loaded with erythromycin (100 mg/mL). To confirm the absence of co-existing un-experimental *S. mutans*, saliva from all animals was collected with sterile cotton swabs and plated on MSB agar plates. To support the implantation of wild and ΔmltG *S. mutans*, rats in both experimental groups were fed a sucrose-enriched (5%) diet ad libitum throughout the experiment. On day 4th, the animals in first two groups were orally inoculated with 200 µL of wild and ΔmltG *S. mutans* (1.4 × 1010 CFU/mL) suspension onto respective animal molar surfaces, once per day for 5 consecutive days to allow oral colonization. 1st Group was inoculated with wild-type, whereas, 2nd group with ΔmltG. On the 11th day, animals were screened for successful infection being developed in the 1st and 2nd groups by oral swabbing and plating as described above. After inoculations, all animals were fed with their respective (i.e., sucrose enriched and normal) diets for the next 10 days to allow the maturation of dental plaque on the animal’s teeth to a detectable extent. The experiments were ended on the 21st day by sacrificing and extracting the lower jaws of animals for biofilm and DCs visualization. All jaws were de-fleshed and suspended in 3.7% formaldehyde until examined under SEM (Miller et al. 2015; Hasan et al. 2015; Kajfasz et al. 2009).

### Comparative ex-situ biofilm formation on a human tooth model

The difference in intrinsic ability of wild and knockout *S. mutans* to form biofilm was further investigated by employing ex-situ biofilm formation on human tooth model. Briefly, human molar teeth were extracted from informed outpatient department patients of Dr. Ziauddin Ahmad Dental College and Hospital (DZADC & H), Aligarh Muslim University, India. The teeth of interest were washed with H_2_O_2_ (6%), then examined under a 10 × stereomicroscope to confirm the removal of soft tissues and stored at 4 °C in a 1% sodium azide solution. Prior to the experiment, the teeth were surface sterilised under UV radiation exposure for 4 h. Thus, prepared teeth were mounted into autoclaved agar in 6 well plates. The wells dedicated to wild and knockout *S. mutans* were treated with THB culture media containing ~ 10^7^ CFU/mL of their respective cells. Whereas, uninfected control teeth were given pristine media at 37ºC and 120 rpm/min. After 72 h of incubation, culture media were discarded, and teeth were washed with sterilised PBS to remove planktonic bacterial cells. Surface intact biofilms on teeth were fixed in 2.5% glutaraldehyde for 6 h at 4ºC. The teeth were treated with alcohols of different dilutions (30, 50, 70, and 90%) for dehydration of the biofilm matrix, and finally were coated with gold film by using sputtering. The SEM images were recorded in the range of 500–3000 × magnification by using SEM (JEOL, Japan) (Forssten et al 2010; Tang et al. 2003).

### Statistical analysis

Statistical analysis was performed by one-way analysis of variance (ANOVA) using the Holm-Sidak method with multiple comparisons with the control group (Sigma Plot 11.0, USA). The level of statistical significance chosen was *p < 0.05, unless otherwise stated. Data were presented in as averaged values of at least three independent experiments done in triplicate.

## Results

### Deletion of mltG induced enhancement in antibiotic susceptibility in *S. mutans*

The deletion of the mltG gene enhanced the susceptibility of ΔmltG type *S. mutans* to the antibiotics, viz., vancomycin, gentamicin, and ciprofloxacin, which primarily act as potential inhibitors for the biosynthesis of bacterial (i) cell-wall, (ii) cytoplasmic proteins, and (iii) DNA, respectively. Data presented in Table 2 exhibits a clear-cut decline in the MICs of vancomycin, gentamicin, and ciprofloxacin at 78, 19, and 312.5 µg/mL against the ΔmltG type of *S. mutans* as compared to the wild type, which was found to be 312.5, 312.5, and 625.0 µg/mL, respectively. The enhanced susceptibility trends in ΔmltG *S. mutans* cells signified cell envelope as the key player in tolerance of wide range of antimicrobials.

**Table 2:** List of primers (with sequences) used in cloning, confirmation of mltG gene deletion and qRT-PCR analyses

Function	Primer name	Forward sequence (5′ → 3′)	Reverse sequence (3′ → 5′)
Cloning	mltG-up	*TACTCGAGATGCTGTCTATGTCTTTATG*	*ATCTGCAGCATAACAGTTCGTGTTTCTT*
	mltG-dw	*ATGGATCCGATGATCTTTATTTTGTAGCC*	*ATGTCGACTATTTTCTACGTCTACGCTA*
	Kan	*ATAGGAGGGATTTATATGAGCCATATTCAA*	*TTAGAAGAACTCATCCATGGATGTCTGGAG*
Transformation analysis	mltG	*ATACTCGAGATGAAAAAGGCTAAGCAATC*	*ATTGGATCCTTACGAATCACTGCTTGA*
qRT-PCR	fruA	*AGCAGATCAAACTACAGAGCCTACTG*	*GGACTGCTCGCACCATCA*
	gtfC	*GGTTTAACGTCAAAATTAGCTGTATTAGC*	*CTCAACCAACCGCCACTGTT*
	clpA	*TTTTGGGAGGCCTGTTGCT*	*TGGCAACGGAGGCAATAATC*
	spaP	*GACTTTGGTAATGGTTATGCATCAA*	*TTTGTATCAGCCGGATCAAGTG*
	vic	*TGACACGATTACAGCCTTTGATG*	*CGTCTAGTTCTGGTAACATTAAGTCCAAT*
	atpA	*TCTGCCCGAGAAAGATCGA*	*GACCATTGTTGCGGATTCG*
	atpC	*TGGAATGGGATCGGACTTTTT*	*TCCAACCCACGTAATTCAAAGG*
	ropA	*GCGGTCGCTAATGCTGAAAT*	*CACGTTGGACCTCATCATGAA*
	comCD	*ACAATTCCTTGAGTTCCATCCAAG*	*TGGTCTGCTGCCTGTTGC*
	ccpA	*GCCAACTCATCCTCAGCAACA*	*GCGCAGCGTGTCATTAATTC*
	ftsX	*GCAGCAAGATTTGGATTG*	*GCTTGGGTTGGTCTTATT*
	murE	*TGCCGACCAGCCACATGATTTC*	*AGGCAGCAGCAACTGCATTTTC*
	murN	*GAGGCTGGCAATCATAAA*	*CGCAGAGAATACAGTGATAA*
	gbpB	*ATGGCGGTTATGGACACGTT*	*TTTGGCCACCTTGAACACCT*

### Influence of mltG deletion on growth dynamics and survival of *S. mutans*

**Fig. 2:**
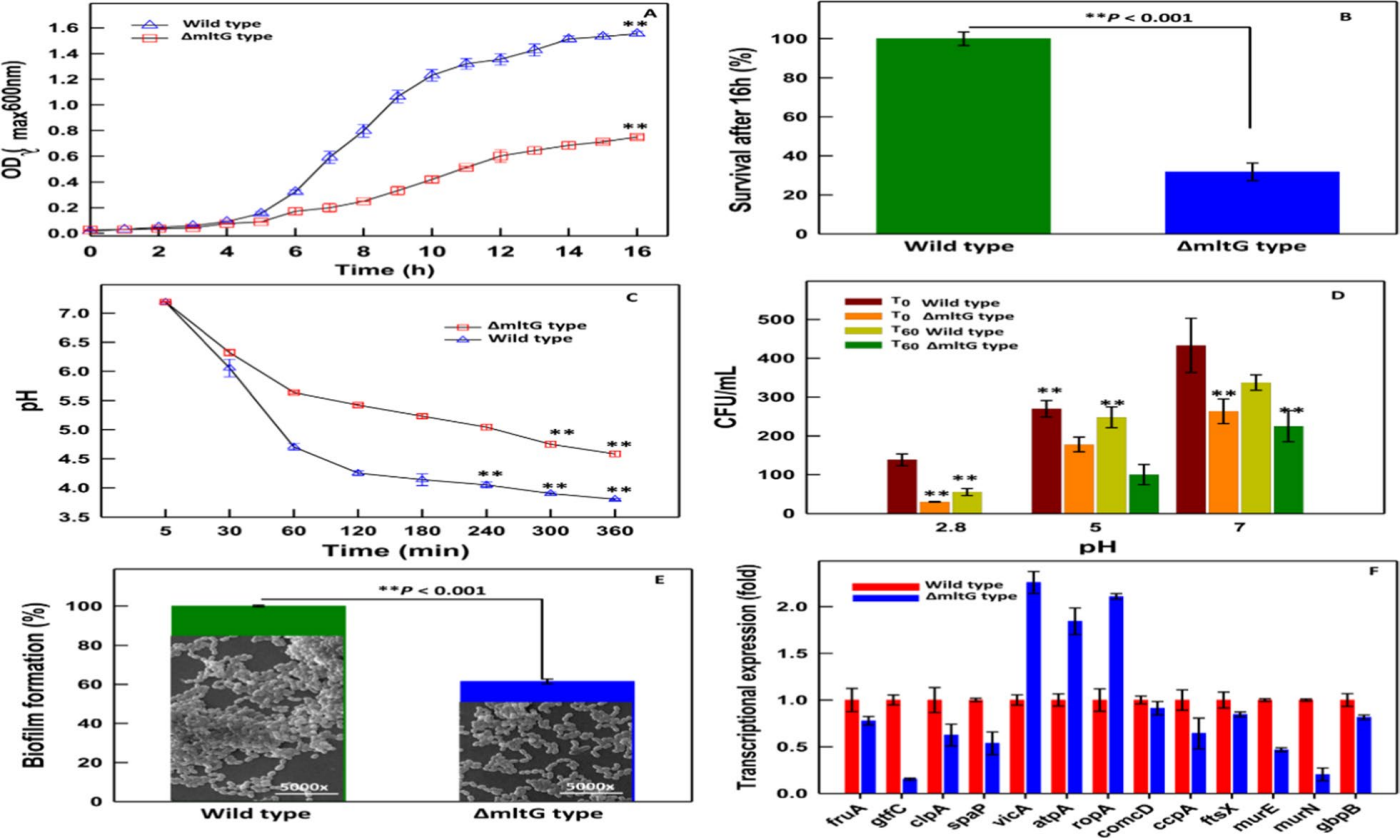
**A** Growth curves of ΔmltG type (indicated with blue line) and wild type (indicated with grey line) strains of *S. mutans*. **B** Survival rate of ΔmltG type with respect to the wild-type has been shown with bar-graph, a significant (p < 0.001) reduction in survival of ΔmltG *S. mutans* was also estimated as 68.2 ± 4.5%. **C** Glycolytic curve. Effect of deletion of mltG gene on acidogenicity of *S. mutans*. Glycolytic acid production was determined by monitoring the pH decrease in glucose solution (1% v/v) over a period of 6 h. Wild-type represented with grey line and ΔmltG type with blue line. **D** Effect of deletion of mltG gene on aciduricity of *S. mutans*. Acid tolerance was determined by measuring the survival rate of *S. mutans* at different pH values such as 2.8, 5.0, and 7.0 on Todd Hewitt agar plates incubated for 48 h at 37˚C. Wild-type represented with grey line and ΔmltG type with blue line. Number of colonies survived expressed in cfu/mL. **E** Biofilm formation by ΔmltG type (represented with blue bar) and wild-type (represented with grey bar) strains of *S. mutans* quantified by crystal violet staining. Results are expressed as means ± standard deviations of triplicate assays from two independent experiments. Image inserts show SEM analysis of of biofilm formed by wild and ΔmltG type strains of *S. mutans*. Scale bar = 10 μm. **F** Quantitative real time qRT-PCR analysis of specific genes to figure out differentially expressed genes in ΔmltG type. Housekeeping gene 16S rRNA were used for normalization. The expression level of the wild-type is set to one for each gene. Significantly up regulated than wild-type (P < 0.05). Significantly down regulated than wild-type (P < 0.05). The assays were performed in triplicate and the means ± SD from three independent experiments were calculated

In determining the impact produced by the loss of the mltG gene on *S. mutans* growth rate, we explored the growing pattern of ΔmltG by growth-curve assay in liquid medium. The results in Fig. 2A demonstrate that deletion of the mltG gene could reduce the growth rate of ΔmltG *S. mutans* significantly (p 0.001) at different points of time as compared to the wild-type strain. With respect to the wild type, a significant (p 0.001) reduction in the survival of ΔmltG *S. mutans* was also estimated as 68.2 ± 4.5% (Fig. 2B). Besides, log phases, i.e., 3–10 h and 5–12 h, were considered to estimate the Td of wild and ΔmltG types of *S. mutans* cells, respectively, by fitting an exponential equation (Y = aebx). Precisely, we observed that mltG deficiency greatly enhanced the Td of the mltG type to 2.72 h (R2 = 0.962) as compared to the wild *S. mutans* cells at 1.48 h (R2 = 0.965). Growth was observed up to 20 h, though both types of bacterial cells, i.e., the parental and the mutant types, reached the stationary phase after 16 h. These results indicate a critical involvement of the mltG gene not only in cell-wall synthesis but also in several growth-promoting and cell division-associated events.

### Glycolytic pH drop and acid killing assay

Considering the fact, we strived to explore the influence of the presence and absence of mltG gene in *S. mutans* over glycolysis, a sugar metabolic pathway, by measuring the glycolytic and fermentative enzymatic efficiencies up to 6 h under identical experimental conditions. The data in Fig. 2C indicate significant acid production with a 0.78-unit pH drop in the case of wild-type *S. mutans* as compared to ΔmltG *S. mutans* (p < 0.001) after 6 h. This decline trend in pH values reflected a tight-coupled relationship between mltG gene and enzymatic activity of glycolytic enzymes of bacteria. (Hasan et al. 2014; Ban et al. 2012). In fact, a drop in pH value indicates the mitigated aciduric potential as well. Nevertheless, in parallel, we also observed a significant (p < 0.001) decrease in CFU in both wild type and ΔmltG *S. mutans* cells, as the pH was shifted from alkaline to an acidic range i.e., pH 7.0 > , 5.0 > , and > 2.8; however, the effect was more pronounced in the constructed mutant type than that of the wild type (Fig. 2D). Besides, 100% elimination of mutant type at acidic pH 2.8 when exposed for 60 min indicates a significantly (p < 0.001) reduced aciduricity of ΔmltG *S. mutans* cells. Taken together, the trends obtained from the production and tolerance of acidic stress, the role of mltG can be envisioned as having involvement in various metabolic pathways that offer a prolonged acid tolerance and survival to aggravate pathogenesis.

### Loss of mltG impairs the propensity of *S. mutans* to adhere, form biofilm and dysregulate the expression of critical genes

Biofilm-like phenotypes formed by the isogenic strains were quantified and compared. To estimate the variance in the biofilm formation between two different strains, a crystal violet assay was carried out. ΔmltG exhibited 40% reduced biofilm formation as compared to the wild type after 48 h of growth, indicating their reduced propensity to form biofilm (Fig. 2E). Also, 52% less tendency for adherence of the ΔmltG type with respect to the wild type was seen. The outcome of the mltG deficiency on *S. mutans* biofilm architecture was also evident when assessed through scanning electron microscopy (SEM) (image inserts in Fig. 2E). qRT-PCR was performed to comprehend the effect produced by mltG on the expression level of virulence genes, viz. fruA, gtfC, clpA, spaP, vicA, atpA, ropA, comcD, ccpA, ftsA, murE, murN, and gbpB. qRT-PCR-based evaluation of the transcriptional expression of biofilm integrity-strengthening genes found fruA, gtfC, gbpB, and comcD in ΔmltG *S. mutans* to be down-regulated at 0.77 ± 0.04, 0.15 ± 0.01, 0.81 ± 0.02, and 0.91 ± 0.07-fold, respectively, compared to wild-type (1.0-fold) (Fig. 2F). The results evidently supported the involvement of mltG gene in structural integrity of biofilm matrices through bio-synthesis of (i) glucan, (ii) glucan-binding protein synthesis, (iii) quorum sensing inducer molecules and (iv), surface adhesion promotion proteins (Krzyściak et al. 2014; Senadheera et al. 2005; Senadheera et al. 2007; Ahn et al. 2008). In particular, comCD is involved in quorum sensing, while gbpB, despite being called a glucan-binding protein, has a primary role in cell-wall metabolism. Similarly, the down-regulation of ftsX, murE, and murN genes in ΔmltG *S. mutans* as 0.84 ± 0.02; 0.46 ± 0.02; and 0.20 ± 0.06-fold (Fig. 2F), respectively, indicates a significant level of impairment in cell division-promoting sub-cellular events and thus delays the Td to 2.72 h, compared to the wild-type (Td = 1.48 h), as described above (Senadheera et al. 2005; Ahn et al. 2008; Liu et al. 2014); Besides, down-regulation of the ccpA and clpA genes in mutant *S. mutans* cells has confirmed the involvement of mlgG in carbohydrate and protein metabolism, respectively (Kajfasz et al. 2009; Cai et al. 2012).

### Influence of mltG deletion over elongasome and divisome components (lipid, protein, and DNA) syntheses in planktonic and biofilm-embedded *S. mutans* cells by flow cytometry-based studies

**Fig. 3:**
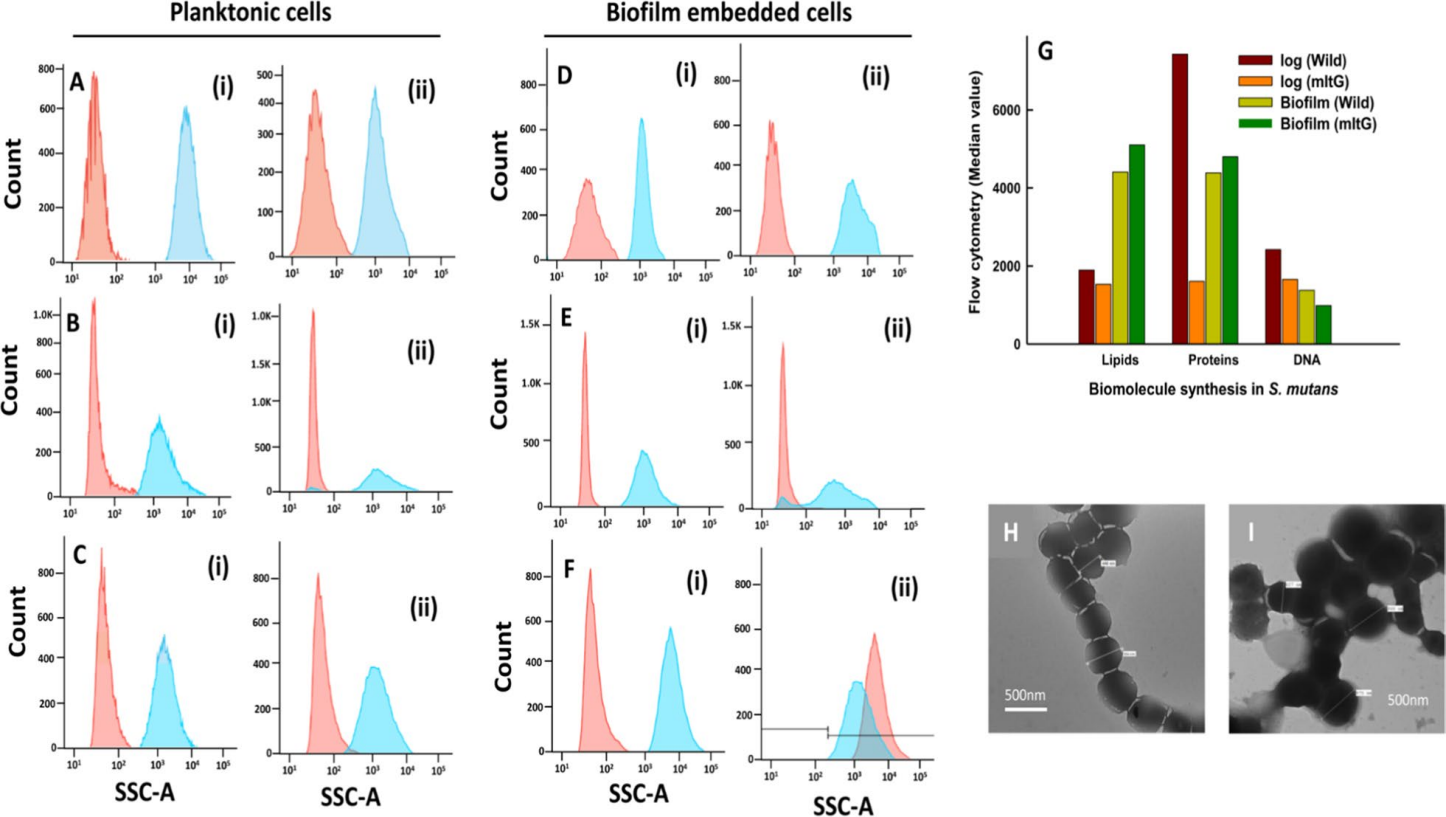
Protein, DNA and Lipid content of wild-type and mltG-mutant were assessed through flow cytometry and represented through histogram overlay in **A**–**C** (log phase metabolic cells), and panels **D**–**F** (biofilm embedded cells) respectively, where panels i & ii represent the wild-type and mutant-type, respectively. Panel **G** exhibits median values of the flow cytometry data presented in panels **A**–**F**. The TEM micrographs of wild (**H**) and mltG (**I**) type *S. mutans* demonstrate difference in size and shape

The median values obtained using flow-cytometry analysis data (Fig. 3B-ii and E-ii) indicated that, compared to wild type, in log-phase growth and biofilm stage, the synthesis of DNA was down-regulated by 762 and 393 median units, respectively (Fig. 3G). Similarly, the deletion of mltG reduced the protein (5818 median units) and lipid (364 median units) in log-phase metabolically active *S. mutans* cells (Fig. 3G), whereas in the case of biofilm, wild, and mltG, an increase in protein (416 median units) and lipid (695 median units) was observed (Fig. 3G). Overall, these results indicate that mltG gene deletion influenced the nexus of several biomolecules involved in elongasome and divisome functions during cell morphogenesis.

### In-vivo caries and ex-situ dental plaque formation assessment

**Fig. 4:**
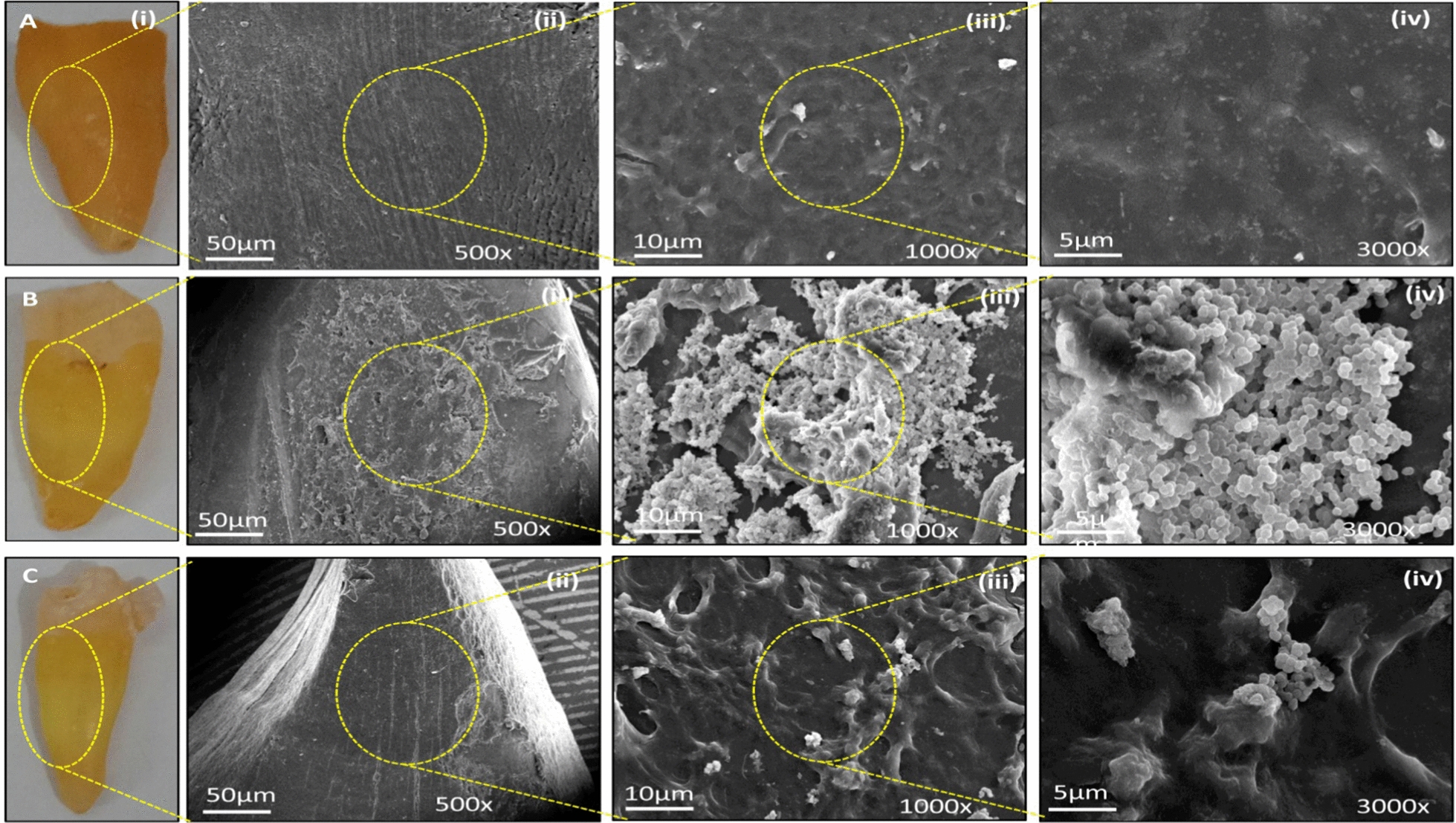
Scanning electron micrographs of extracted human teeth. SEM analysis of extracted human teeth to evaluate the level of caries developed by wild-type and ΔmltG-type

**Fig. 5:**
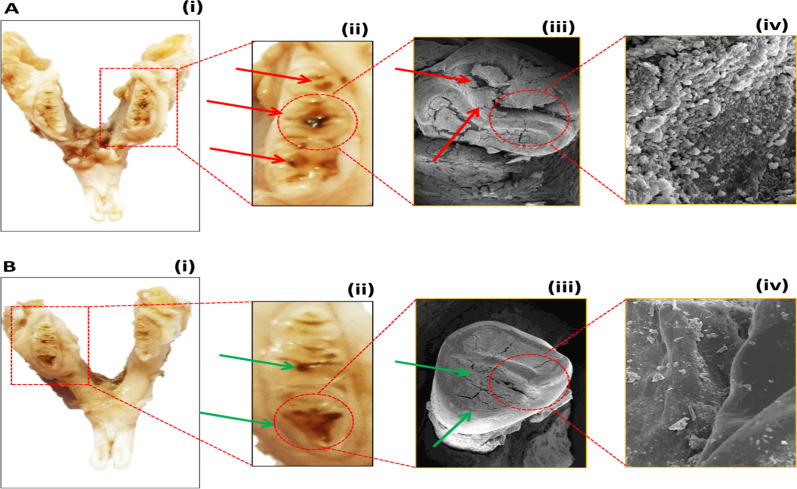
Scanning electron micrographs of aseptically removed rat teeth. SEM analysis of rat’s teeth to evaluate the level of caries developed by wild-type and ΔmltG-type

The formation of bacterial biofilm, plaque, and caries in the presence of natural body conditions such as body temperature and salivary pH in the oral cavity offer a complex bacteria-dentin interaction landscape. Nevertheless, in rat’s oral cavity experiments, a prolonged exposure of wild and ΔmltG *S. mutans* and sugar enriched diet intake could create distinct levels of dentin lesions and plaque formation. Concisely, the SEM analysis of the rats’ molar teeth clearly depicted the demineralization of the enamel surface of teeth with caries progression up to the dentin layer, thereby indicating the formation of well-established caries in the group infected with wild-type (Fig. 4Ai). The surface has an evident biofilm embedded in the EPS pool (Fig. 5Aii–iv), whereas the group treated with ΔmltG *S. mutans* exhibited comparatively shallow caries and depleted biofilm matrix formation, as demonstrated in (Fig. 5Bi–iv), respectively. Overall, SEM based DCs and biofilm observations were in good agreement with mltG dependent multifaceted virulence promoting activities of *S. mutans* obtained from the systematic comparative *in-vitro* studies. Colony forming unit (CFU) were also performed to estimate the proportion of wild type and ΔmltG *S. mutans* on 11th and 21st day on rat’s teeth provided in the Additional file 1: Fig. S3.

## Discussion

Endolytic murein-transglycosylase (mltG) is an inner membrane enzyme that belongs to the class of lytic transglycosylases. mltG possesses endolytic transglycosylase activity; hence, it acts as a terminase to endolytically control the PG strand elongation. LTs-mediated PG remodelling was recently highlighted by Winther et al. 2021, who showed the interaction between RNA-binding proteins EloR and mltG at midcell in *S. pneumoniae* R6. Whereas, Bohrhunter et al. 2021, have also shown the antagonistic potential of mltG against cell-wall synthesis by both PG polymerases in *E. coli*. In fact, the inactivation or deletion of mltG can adversely suppress the requirement of PBP2b, MreCD, RodA, and RodZ, genes that are involved in *S. pneumococcal* elongation machinery (Tsui et al. 2016). However, the role of mltG in the virulence of bacteria is still an untouched topic, as long as we review the available literature. Here we evaluated whether mltG is involved in regulating the overall physiology and different virulence attributes of *S. mutans*, which has not been documented hitherto in any bacteria. This study has presented *in-vitro*, *in-vivo*, and *ex-vivo* experimental findings to demonstrate that loss of ΔmltG resulted in lower virulence potential in comparison to the wild-type strain. On the basis of our findings, it can be inferred that mltG gene products modulate susceptibility to antibiotics, adherence, biofilm formation, growth rate, acidogenicity, and aciduricity in *S. mutans*. When the MIC of the constructed mutant strain, i.e., ΔmltG was evaluated, it exhibited an augmented sensitivity to antibiotics such as vancomycin, gentamycin, and ciprofloxacin as compared to the wild type. Enhancement in susceptibility of ΔmltG type against vancomycin, as 78 µg/mL, compared to wild counterpart as 312.5 µg/mL, indicated that the absence of mltG gene ameliorated the activity of LTs in the cell-wall PGs synthesis and remodelling. Consequently, mltG deficient cells may experience the lack of remodelled or charged PGs building blocks to develop rigid bacterial cell envelope to evade the antimicrobial stress during the cell proliferation (Suntharalingam et al. 2009). This mltG gene induced disruption in the bio-synthesis of PGs building blocks can be speculated to lead a compromised cell-wall structure in ΔmltG *S. mutans* with increased permeability for antimicrobials and extracellular entities influx. Nevertheless, after cell-wall disruption mediated vancomycin killing, we envisioned an uninterrupted influx of antimicrobials into the cytoplasm of ΔmltG *S. mutans* cells. Surprisingly, antibiotics interfering in protein and DNA synthesis, such as gentamicin and ciprofloxacin, could effectively reduce the viability (~ 80–90%) of ΔmltG *S. mutans* at MICs of 19 and 312.5 µg/mL, compared to wild type MICs of 312.5 and 625.0 µg/mL, respectively (Table 2). Importantly, rapid proliferation and clustering of pathogenic bacteria can play a decisive role in virulence aggravation in several clinical and biomedical settings. For instance, demineralization of tooth surfaces largely depends on the robust metabolic activities (e.g., production of EPS and organic acids) of rapidly growing and clustering *S. mutans* cells, which cover the dentin and their ecological niche so as to create dental caries or lesions in depth (Niu et al. 2021). This scenario indeed prompted us to investigate the influence of mltG deletion on the (i) growth dynamics, (ii) survival, and (iii) generation doubling time (Td) of ΔmltG *S. mutans* and wild-type cells under identical experimental conditions, i.e., culture media (THB), temperature (37 °C), agitation (120 rpm), and incubation time (0–20 h). In this line, the study by Perez et al. 2018, has demonstrated that cell division and PG synthesis in *Streptococcus species* are orchestrated by the coordinated dynamic movement of essential protein complexes. Hence, the deletion of mltG can be envisioned to negatively induce the cellular events that are tightly associated with mltG regulations in *S. mutans* cells, which leads to a reduced growth rate. Aciduric nature is another strong feature involved in the virulence of *S. mutans*. Due to this factor, *S. mutans* is able to perform glycolysis at markedly low pH values. In view of this fact, they are capable of maintaining pH homeostasis across the membrane of the bacterial cell so as to regulate cytoplasmic alkalinity (Xu et al. 2011). Thus, their suppressed acid-producing capability and aciduricity may lead to the impaired functioning of an array of enzymes regulating diverse physiological processes such as reduced glycolytic efficacies of microbial cells, dysregulation of cell persistence, obstruction in IPS (intracellular polysaccharides), and EPS production, thereby causing the potential mortality of this cariogenic bacteria (Hasan et al. 2014). Similarly, the study of Hasan et al. 2014, demonstrated that the inhibition of major virulence pathways can trigger the impairment of the enzymatic potential of a series of enzymes associated with physiological processes (such as glycolysis, and intra- and extracellular polysaccharide production) related to the virulence aggravating vigour of *S. mutans*. Nevertheless, in view of our results, the role of the mltG gene, particularly in *S. mutans* is associated dental caries virulence control, can be strongly advocated. Considering the fact that, in comparison with cell suspensions, multi-component biofilm-embedded *S. mutans* cells do not behave identically when exposed to antimicrobial agents, we found it interesting to assess mltG gene involvement in biofilm formation and surface adherence.

The data in Fig. 2E exhibit a 40% reduced propensity for biofilm formation in ΔmltG *S. mutans* as compared to wild-type (p < 0.001). SEM-based parallel investigation of biofilm adherence on a glass surface also validated our results, demonstrating sparsely distributed biofilm architecture produced by ΔmltG *S. mutans* as compared to wild-type cells (image inserts in Fig. 2E). Considering more than 52% reduced efficiency of ΔmltG *S. mutans* cells to adhere to glass surfaces prompted us to explore the association of mltG with transcriptional expressions of co-existing virulence genes. Genes important for biofilm formation like, fruA, gtfC, spaP, comCD, gbpB, genes like clpA and ccpA involved in protein and carbohydrate metabolism respectively, as well as cell divison genes like ftsX, murE, murN were found many folds supressed in the mutant type. However, stress-responsive genes like vicA, ropA, and atpC were found overexpressed in ΔmltG, indicating the augmented sensing of environmental stress (Fig. 2F). Nevertheless, in view of obtained trends from mutant and wild *S. mutans*, we can conclude that our presumption to circumvent the virulence of *S. mutants* in plaque formation and dental cariogenesis though mltG deletion has provided significant insights which are highly relevant in advanced state-of-the-arts required in clinical management of *S. mutants* associated inflations. Indeed, during cell morphogenesis, particularly in *Streptococcus* species, bacterial cells create a protective layer of PGs to envelop the cells to sustain a synchronous synthesis of essential biomolecules, including lipids, proteins, carbohydrates, and nucleic acids, to function as elongasome and divisome complexes accordingly. Particularly, proteins like RNA-binding and Ser/Thr kinase in elongasome coordinated protein complex control cell elongation. But, several elongasome proteins have been found interacting with the LTs homolog mltG (Perez et al. 2018). At another end, the divisome triggers the synthesis of essential components to create a septal disc that facilitates binary fission in bacterial cells. At the same time, divisomes also play a central role in the scaffolding, localization, and regulation of PG production across the cell envelope to create division zones so as to regulate the elongation and division of bacterial cells (Winther et al. 2021 ; Egan et al. 2020). Flow cytometric analysis was carried out to measure and compare the components of elongasome and divisome (lipids, proteins and DNA) of log phase- wild type and mltG deficient strain cells (during which elongasome and divisome are most active). Since, elongasome and divisome and non-functional during biofilm stage, biofilm embedded cells were taken to determine the overall change in median values of lipids, proteins and DNA of the whole cell, so as to get the idea that variations in the median values in log phase is majorly due the changes in the components of elongasome and divisome (Bjarnsholt et al. 2013). As evident from the results of the flow cytometric analysis, maximum variance in the components between wild and ΔmltG type was found for log phase cells, thereby giving the idea of obstructed elongasome and divisome activity in mutant cells due to the altered ratio of their three components (lipids, proteins and DNA). In this context, Besides, we speculate that deletion of the mltG gene in *S. mutans* leads to improper cell division, which was also validated through suppressed expression of cell division genes like ftsX, murE, and murN. Defective cell division causes enlargement of cell size, as observed under transmission electron microscopy. TEM-based size analysis also demonstrated a significant change in diameter as 627–675 nm and 448–554 nm in ΔmltG *S. mutans* (Fig. 3I) and wild-type cells (Fig. 3H), respectively. Moreover, in spherically shaped bacteria such as *S. mutans*, inhibition of DNA synthesis leads to the inhibition of cell division and an increase in cell surface area (Higgins et al. 1974). The ex-situ human tooth and Wistar rat model were employed to assess the influence of mltG over plaque formation and cariogenic potential, respectively. The SEM analysis of human incisor teeth exposed to wild-type (Fig. 4Bi–iii) and ΔmltG *S. mutans* (Fig. 4 Ci–iii) evidently supported the ΔmltG mediated impairment of biofilm formation on the teeth’s enamel surface. A clear-cut reduction in ΔmltG *S. mutans* density while biofilm formation progressed is in good agreement with our mltG-mediated biofilm ameliorating trends, as discussed above.

The present study demonstrates the systematic, development of the mltG deficient gene construct in *S. mutans* and the evaluation of the virulence of wild and ΔmltG *S. mutans* under three different biofilm models, including (i) *in-vitro* glass surface adherence and (ii) ex-situ human tooth and (iii) *in-vivo* animal molar tooth models for biofilm and cariogenic lesions. Preliminary results showed that *S. mutans* virulence-associated activities such as cell division, Td, acid tolerance, metabolism of sugars to produce organic acids and EPS, and components of divisome and elongasome were significantly deregulated in ΔmltG *S. mutans* as compared to the wild type, particularly for log phase cells. Next, perturbation in the transcriptional expressions of *S. mutans’* genes, which solely contribute to biofilm formation, quorum sensing, EPS production, and cell-wall building block synthesis, has warranted the association of mltG with the nexus of complex metabolic systems. Besides, mltG deletion-based mitigation in virulence of *S. mutans* in dental settings, that is, human and rodent’s dentin lesions demonstrated a clear-cut compromised tooth infection trend in the case of ΔmltG *S. mutans*, compared to the wild type counterpart. Overall, these results provided significant insights that can effectively encourage the research developments progressing against *S. mutans* associated virulence havoc to consider the mltG gene as a potential therapeutic target.

## Supplementary Information

**Additional file 1: Fig. S1.** Sequence data obtained for wild type strain, when balst was performed for forward and reverse orientations, 99% homology was matched with mltG gene of S. mutans. **Fig. S2.** ΔmltG type type searched for forward orientation: Sequence data obtained for mltG deficient strain,when balst was performed for forward orientation and reverse orientation, 99% homology was matched with vectors containing kanamycin casette for selection. Kanamycin cassette replaced the mltG gene by homologous recombination and got incorporated in its place. **Fig. S3.** Colony forming unit to check the proportion of wild type and ΔmltG S. mutans on rat’s teeth.

## Data Availability

All data is available as main figures and table in manuscript as well as supplementary data attached.

## References

[CR1] Ahn SJ, Wen ZT, Burne RA (2006). Multilevel control of competence development and stress tolerance in *Streptococcus mutans* UA159. Infect Immun.

[CR2] Ahn SJ, Ahn SJ, Wen ZT, Brady LJ, Burne RA (2008). Characteristics of biofilm formation by *Streptococcus mutans* in the presence of saliva. Infect Immun.

[CR3] Ban SH, Kim JE, Pandit S, Jeon JG (2012). Influences of *Dryopteris crassirhizoma* extract on the viability, growth and virulence properties of *Streptococcus mutans*. Molecules.

[CR4] Banas JA, Vickerman MM (2003). Glucan-binding proteins of the oral *streptococci*. Crit Rev Oral Boil Med.

[CR5] Birlutiu V, Birlutiu RM, Costache VS (2018). *Viridans streptococcal* infective endocarditis associated with fixed orthodontic appliance managed surgically by mitral valve plasty: a case report. Medicine.

[CR6] Bjarnsholt T, Ciofu O, Molin S, Givskov M, Høiby N (2013). Applying insights from biofilm biology to drug development—can a new approach be developed?. Nat Rev Drug Discov.

[CR7] Bohrhunter JL, Rohs PD, Torres G, Yunck R, Bernhardt TG (2021). MltG activity antagonizes cell wall synthesis by both types of peptidoglycan polymerases in *Escherichia coli*. Mol Microbiol.

[CR8] Cai J, Tong H, Qi F, Dong X (2012). CcpA-dependent carbohydrate catabolite repression regulates galactose metabolism in *Streptococcus oligofermentans*. J Bacteriol.

[CR9] Egan AJ, Errington J, Vollmer W (2020). Regulation of peptidoglycan synthesis and remodelling. Nat Rev Microbiol.

[CR10] Fejerskov O, Kidd E (2008). Dental caries: the disease and its clinical management.

[CR11] Forssten SD, Björklund M, Ouwehand AC (2010). *Streptococcus mutans*, caries and simulation models. Nutrients.

[CR12] Gregoire S, Singh AP, Vorsa N, Koo H (2007). Influence of cranberry phenolics on glucan synthesis by glucosyltransferases and *Streptococcus mutans* acidogenicity. J Appl Microbiol.

[CR13] Guevara MA, Lu J, Moore RE, Chambers SA, Eastman AJ, Francis JD, Noble KN, Doster RS, Osteen KG, Damo SM, Manning SD (2020). Vitamin D and *Streptococci*: the interface of nutrition, host immune response, and antimicrobial activity in response to infection. ACS Infect Dis.

[CR14] Guo L, McLean JS, Lux R, He X, Shi W (2015). The well-coordinated linkage between acidogenicity and aciduricity via insoluble glucans on the surface of *Streptococcus mutans*. Sci Rep.

[CR15] Hamada S, Slade HD (1980). Biology, immunology, and cariogenicity of *Streptococcus mutans*. Microbiol Rev.

[CR16] Hasan S, Singh K, Danisuddin M, Verma PK, Khan AU (2014). Inhibition of major virulence pathways of *Streptococcus mutans* by Quercitrin and Deoxynojirimycin: a synergistic approach of infection control. PLoS ONE.

[CR17] Hasan S, Danishuddin M, Khan AU (2015). Inhibitory effect of zingiber officinale towards *Streptococcus mutans* virulence and caries development: in vitro and in vivo studies. BMC Microbiol.

[CR18] Higgins ML, Daneo-Moore L, Boothby D, Shockman GD (1974). Effect of inhibition of deoxyribonucleic acid and protein synthesis on the direction of cell wall growth in *Streptococcus*
*faecalis*. J Bacteriol.

[CR19] Jorgenson MA, Chen Y, Yahashiri A, Popham DL, Weiss DS (2014). The bacterial septal ring protein RlpA is a lytic transglycosylase that contributes to rod shape and daughter cell separation in *Pseudomonas aeruginosa*. Mol Microbiol.

[CR20] Kajfasz JK, Martinez AR, Rivera-Ramos I, Abranches J, Koo H, Quivey RG (2009). Role of Clp proteins in expression of virulence properties of *Streptococcus mutans*. J Bacteriol.

[CR21] Kaspar JR, Lee K, Richard B, Walker AR, Burne RA (2021). Direct interactions with commensal streptococci modify intercellular communication behaviors of *Streptococcus mutans*. ISME J.

[CR22] Knilans KJ, Hackett KT, Anderson JE, Weng C, Dillard JP, Duncan JA (2017). *Neisseria gonorrhoeae* lytic transglycosylases LtgA and LtgD reduce host innate immune signalling through TLR2 and NOD2. ACS Infec Dis.

[CR23] Krzyściak W, Jurczak A, Kościelniak D, Bystrowska B, Skalniak A (2014). The virulence of *Streptococcus mutans* and the ability to form biofilms. Eur J Clin Microbiol Infect Dis.

[CR24] Lee W, McDonough MA, Kotra LP, Li ZH, Silvaggi NR, Takeda Y (2001). A 1.2 Å snapshot of the final step of bacterial cell wall biosynthesis. Proc Natl Acad Sci USA.

[CR25] Lee M, Hesek D, Suvorov M, Lee W, Vakulenko S, Mobashery S (2003). A mechanism-based inhibitor targeting the DD-transpeptidase activity of bacterial penicillin-binding proteins. J Am Chem Soc.

[CR26] Lemos JA, Abranches J, Burne RA (2005). Responses of cariogenic *streptococci* to environmental stresses. Curr Issues Mol Biol.

[CR27] Liu X, Miller P, Basu U, McMullen LM (2014). Sodium chloride-induced filamentation and alternative gene expression of fts, murZ, and gnd in *Listeria monocytogenes* 08–5923 on vacuum-packaged ham. FEMS Microbiol Let.

[CR28] Loesche WJ (1986). Role of *Streptococcus mutans* in human dental decay. Microbiol Rev.

[CR29] Loo CY, Corliss DA, Ganeshkumar N (2000). *Streptococcus gordonii* biofilm formation: identification of genes that code for biofilm phenotypes. J Bacterial.

[CR30] Miglani S (2019). Burden of dental caries in India: Current scenario and future strategies. Int J Clin Pediatr Dent.

[CR31] Miller JH, Avilés-Reyes A, Scott-Anne K, Gregoire S, Watson GE, Sampson E (2015). The collagen binding protein Cnm contributes to oral colonization and cariogenicity of *Streptococcus mutans* OMZ175. Infect Immun.

[CR32] Misba L, Zaidi S, Khan AU (2017). A comparison of antibacterial and antibiofilm efficacy of phenothiazinium dyes between Gram-positive and Gram-negative bacterial biofilm. Photodiagnosis Photodyn.

[CR33] Niu JY, Yin IX, Wu WK, Li QL, Mei ML, Chu CH (2021). Antimicrobial peptides for the prevention and treatment of dental caries: a concise review. Arch Oral Biol.

[CR34] Padfield D, Castledine M, Buckling A (2020). Temperature-dependent changes to host–parasite interactions alter the thermal performance of a bacterial host. ISME J.

[CR35] Perez AJ, Cesbron Y, Shaw SL, Villicana JB, Tsui HC, Boersma MJ (2018). Movement dynamics of divisome and penicillin-binding proteins (PBPs) in cells of *Streptococcus pneumoniae*. bioRxiv.

[CR36] Petersen PE, Bourgeois D, Ogawa H, Estupinan-Day S, Ndiaye C (2005). The global burden of oral diseases and risks to oral health. Bull World Health Organ.

[CR37] Pratt RF (2016). β-Lactamases: why and how. J Med Chem.

[CR38] Sassine J, Pazos M, Breukink E, Vollmer W (2021). Lytic transglycosylase MltG cleaves in nascent peptidoglycan and produces short glycan strands. Cell Surf.

[CR39] Schwendicke F, Dörfer CE, Schlattmann P, Foster Page L, Thomson WM, Paris S (2015). Socioeconomic inequality and caries: a systematic review and meta-analysis. J Dent Res.

[CR40] Segal R, Pisanty S, Wormser R, Azaz E, Sela MN (1985). Anticariogenic activity of licorice and glycyrrhizine I: inhibition of in vitro plaque formation by *Streptococcus mutans*. J Pharm Sci.

[CR41] Senadheera MD, Guggenheim B, Spatafora GA, Huang YC, Choi J, Hung DC, Treglown JS, Goodman SD, Ellen RP, Cvitkovitch DG (2005). A VicRK signal transduction system in *Streptococcus mutans* affects gtfBCD, gbpB, and ftf expression, biofilm formation, and genetic competence development. J Bact.

[CR42] Senadheera MD, Lee AW, Hung DC, Spatafora GA, Goodman SD, Cvitkovitch DG (2007). The *Streptococcus mutans* vicX gene product modulates gtfB/C expression, biofilm formation, genetic competence, and oxidative stress tolerance. J Bacteriol.

[CR43] Silhavy TJ, Kahne D, Walker S (2010). The bacterial cell envelope. Cold Spring Harb Perspect Biol.

[CR44] Suntharalingam P, Senadheera MD, Mair RW, Levesque CM, Cvitkovitch DG (2009). The LiaFSR system regulates the cell envelope stress response in *Streptococcus mutans*. J Bacteriol.

[CR45] Sztajer H, Szafranski SP, Tomasch J, Reck M, Nimtz M, Rohde M, Wagner-Döbler I (2014). Cross-feeding and interkingdom communication in dual-species biofilms of *Streptococcus mutans* and *Candida albicans*. ISME J.

[CR46] Tang G, Yip HK, Cutress TW, Samaranayake LP (2003). Artificial mouth model systems and their contribution to caries research: a review. J Dent.

[CR47] Tsui HC, Zheng JJ, Magallon AN, Ryan JD, Yunck R, Rued BE, Bernhardt TG, Winkler ME (2016). Suppression of a deletion mutation in the gene encoding essential PBP2b reveals a new lytic transglycosylase involved in peripheral peptidoglycan synthesis in *Streptococcus pneumoniae* D39. Mol Microbiol.

[CR48] Valdez RM, Duque C, Caiaffa KS, Dos Santos VR, Loesch ML, Colombo NH (2017). Genotypic diversity and phenotypic traits of *Streptococcus mutans* isolates and their relation to severity of early childhood caries. BMC Oral Health.

[CR49] Viala J, Chaput C, Boneca IG, Cardona A, Girardin SE, Moran AP, Athman R, Mémet S, Huerre MR, Coyle AJ, DiStefano PS (2004). Nod1 responds to peptidoglycan delivered by the *Helicobacter pylori* cag pathogenicity island. Nat Immunol.

[CR50] Vollmer W, Seligman SJ (2010). Architecture of peptidoglycan: more data and more models. Trends Microbiol.

[CR51] Walter A, Mayer C (2019) Peptidoglycan structure, biosynthesis, and dynamics during bacterial growth. In: Extracellular sugar-based biopolymers matrices, Springer, Cham, pp 237–99. 10.1007/978-3-030-12919-4_6

[CR52] Wen ZT, Bitoun JP, Liao S (2015). PBP1a-deficiency causes major defects in cell division, growth and biofilm formation by *Streptococcus mutans*. PLoS ONE.

[CR53] Wientjes FB, Woldringh CL, Nanninga N (1991). Amount of peptidoglycan in cell walls of Gram-negative bacteria. J Bacteriol.

[CR54] Williams AH, Wheeler R, Deghmane AE, Santecchia I, Schaub RE, Hicham S, Nilges MM, Malosse C, Chamot-Rooke J, Haouz A, Dillard JP (2020). Defective lytic transglycosylase disrupts cell morphogenesis by hindering cell wall de-O-acetylation in *Neisseria meningitidis*. Elife.

[CR55] Winther AR, Kjos M, Herigstad ML, Håvarstein LS, Straume D (2021). EloR interacts with the lytic transglycosylase MltG at midcell in *Streptococcus pneumoniae* R6. J Bacteriol.

[CR56] Xu X, Zhou XD, Wu CD (2011). The tea catechin epigallocatechin gallate suppresses cariogenic virulence factors of *Streptococcus mutans*. Antimicrob Agents Chemother.

[CR57] Yunck R, Cho H, Bernhardt TG (2016). Identification of MltG as a potential terminase for peptidoglycan polymerization in bacteria. Mol Microbiol.

[CR58] Zaidi S, Singh SL, Khan AU (2020). Exploring antibiofilm potential of bacitracin against *Streptococcus mutans*. Microb Pathog.

[CR59] Zeng L, Wen ZT, Burne RA (2006). A novel signal transduction system and feedback loop regulate fructan hydrolase gene expression in *Streptococcus mutans*. Mol Microbiol.

