# Supplementary material for: mltG gene deletion mitigated virulence potential of Streptococcus mutans: An in-vitro, ex-situ and in-vivo study
Source: AMB Express. 2023 Feb 20;13:19. doi: 10.1186/s13568-023-01526-x (PMC9941400; doi:10.1186/s13568-023-01526-x)
Supplement: Supplementary file 1 — Additional file 1: Fig. S1. Sequence data obtained for wild type strain, when balst was performed for forward and reverse orientations, 99% homology was matched with mltG gene of S. mutans. Fig. S2. ΔmltG type type searched for forward orientation: Sequence data obtained for mltG deficient strain,when balst was performed for forward orientation and reverse orientation, 99% homology was matched with vectors containing kanamycin casette for selection. Kanamycin cassette replaced the mltG gene by homologous recombination and got incorporated in its place. Fig. S3. Colony forming unit to check the proportion of wild type and ΔmltG S. mutans on rat’s teeth. [file 13568_2023_1526_MOESM1_ESM.pdf]

mltG gene deletion mitigated virulence potential of *Streptococcus mutans*: An *in-vitro, ex-situ* and *in-vivo* study

Sahar Zaidi[a], Khursheed Ali[a], Yadya M Chawla[b] and Asad U Khan[a*]

[a]Medical Microbiology and Molecular Biology, Laboratory Interdisciplinary, Biotechnology Unit, Aligarh Muslim University, Aligarh, 202002, UP, India.

[b]ICGEB-Emory Vaccine Center, International Centre for Genetic Engineering and Biotechnology, New Delhi, India

Running Title: Exploring the role of mltG gene in the virulence of *Streptococcus mutans*.

***Corresponding Author**:
Professor Asad U Khan
Interdisciplinary Biotechnology Unit,
Aligarh Muslim University, Aligarh-202002, UP, India.
Tel: 0091-9837021912
Fax: 0091-571-2721776
(Email: asad.k@rediffmail.com/asadukhan72@gmail.com)

Sequencing data

Fig. S1 Sequence data obtained for wild type strain, when balst was performed for forward and reverse orientations, 99% homology was matched with mltG gene of *S. mutans.*

Fig. S1-a

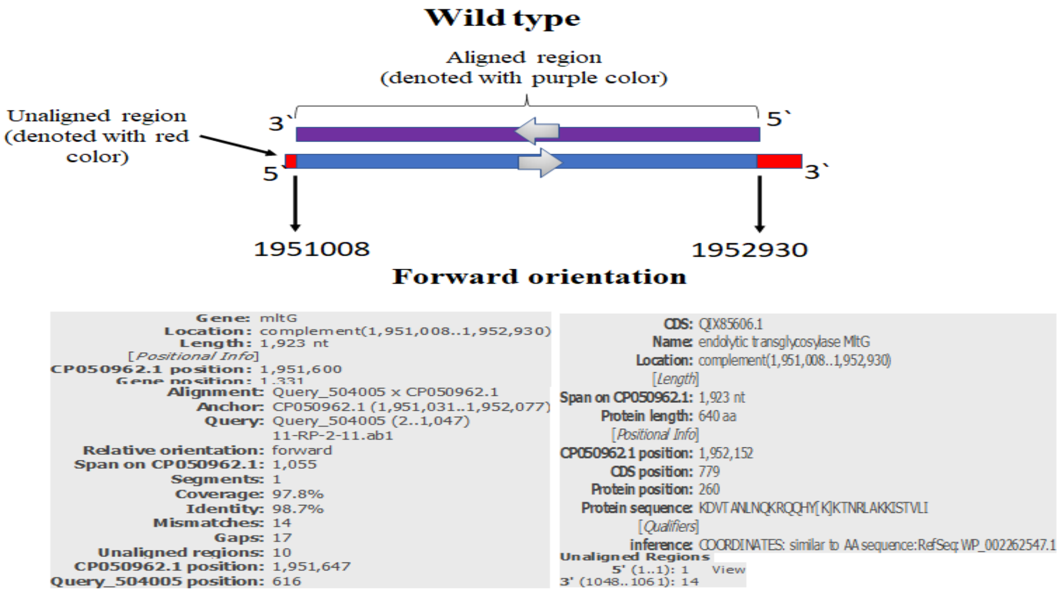

Fig. S1-b

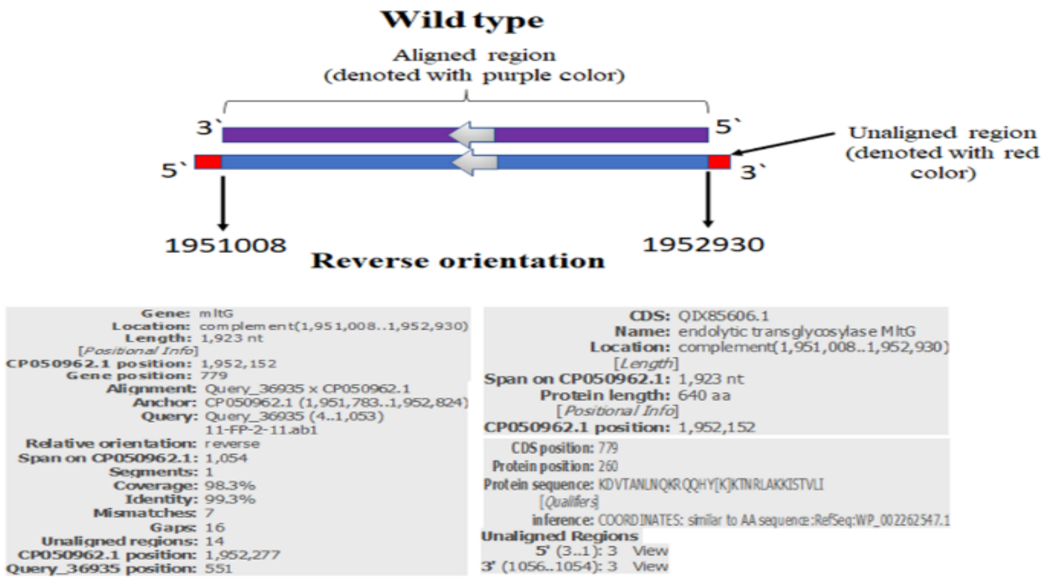

Fig. S2 ΔmltG type type searched for forward orientation: Sequence data obtained for mltG deficient strain, when balst was performed for forward orientation and reverse orientation, 99% homology was matched with

vectors containing kanamycin casette for selection. Kanamycin cassette replaced the mltG gene by homologous recombination and got incorporated in its place.

Fig. S2-a

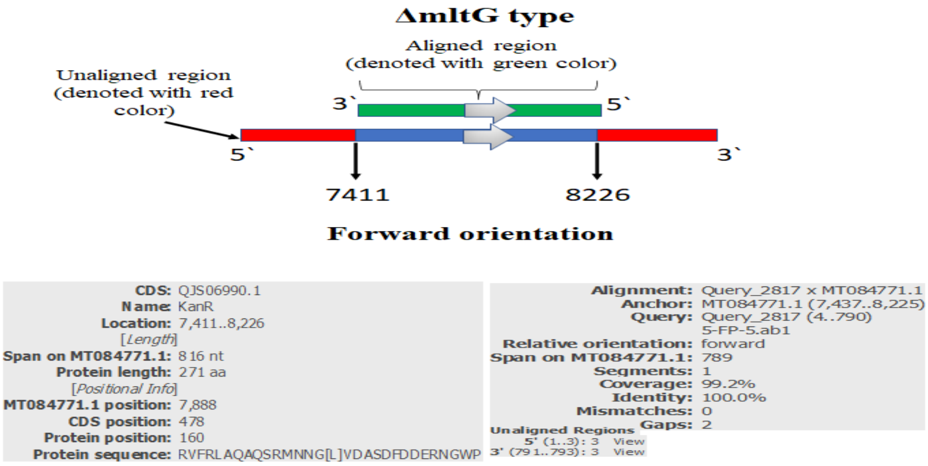

Fig. S2-b

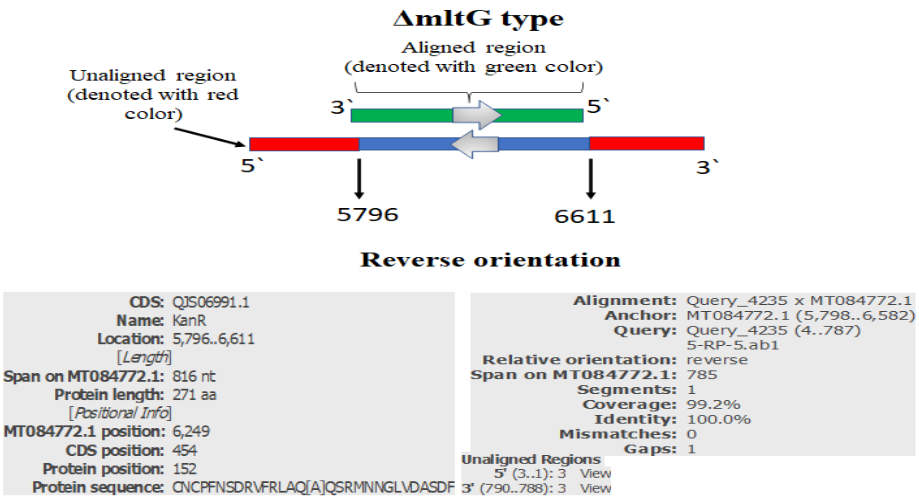

Fig. S3 Colony forming unit to check the proportion of wild type and ΔmltG *S. mutans* on rat's teeth

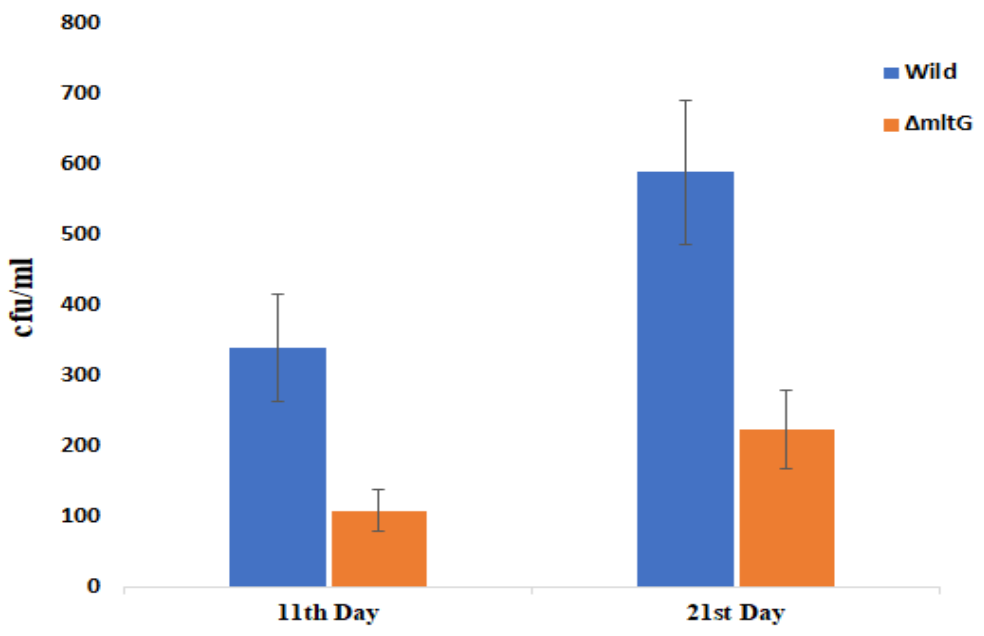

Materials and methods

Flow cytometry

BD X-20 LSR Fortessa flow cytometer was used in this study and sample and data analysis were done with Flowjo 10 software. The instrument was set to detect 10,000 cells (events) per sample. Forward scatter (FSC) and side scatter (SSC) were detected using linear amplification. The DNA, lipid and protein content of cells of log phase and biofilm of *Streptococcus mutans* were measured at a rate of 10^4 cells per second. DNA was quantified by measuring the fluorescence from cells stained with a combination of the DNA binding drugs mithramycin and ethidium bromide and Nile red quantified lipid content of the cell whereas FITC detected proteins. All the dyes purchased from Santa Cruz Biotechnology. Dual parameter histograms (fluorescence/scattered light) of bacterial cultures gave detailed pictures of changes arising as a result of deletion of mltG gene. One of the major drawbacks in applying flow cytometry to bacterial populations is the natural clumping of cells. To overcome this problem, cell chains were disaggregated by a mild sonication procedure prior to flow cytometric analysis, ensuring the assessment of the physiological status on a cell-by-cell basis.

Mithramycin and EtBr for DNA quantification

A biofilm was formed both by wild type and ΔmltG strain in six well plates at 37°C and for 48 hours in Todd Hewitt broth supplemented with 1% sucrose. After incubation, biofilm suspension was transferred to tubes and sonicated to separate the adhered cells of the biofilm. Log phase cells (O.D. = 0.5) of both wild type and ΔmltG

strain were also evaluated for DNA content. Bacterial cells were washed with 0.1M Tris/HCL buffer pH 7.4 and suspended in buffer (containing 100 µg/ml mithramycin, 50 µg/ml EtBr, 25mM $MgCl_2$ and 100mM NaCl dissolved in tris buffer of pH 7.4). The fluorescence of bacterial cells stained by mithramycin and ethidium bromide is closely proportional to their DNA contents. Mithramycin (excitation maximum 400 nm) was thus excited at close to its optimum wavelength but, importantly, ethidium bromide (excitation maximum 493 nm) was not well excited. Under these conditions, DNA-bound ethidium bromide is excited by fluorescence resonance energy transfer from mithramycin. Mithramycin has a high specificity for DNA, but weak fluorescence. In contrast ethidium bromide is strongly fluorescent but binds to other polyanions in the cell. The dye mixture thus gives a valuable combination of high specificity and cells with a detectable fluorescence (99% of unstained cells analysed under the same conditions appeared below fluorescence channel) (Steen et al., 1994; Winson and Davey 2000).

Nile red for lipid quantification / Membrane staining

Log phase as well as sonicated biofilm cells of wild type and ΔmltG strain (OD600nm = 0.3) were stained with 10 µg/ml Nile red (excitation at 515nm) for 30 min at 37◦C. After washing the cells twice with PBS, the bacteria were analysed on flow cytometry using petxrd channel (Sugimoto et al., 2017; Aqawi et al., 2021).

Fluorescein isothiocyanate (FITC) for protein quantification

For protein staining, the bacterial cells (log phase and biofilm cells) were washed once in 0.1 M-phosphate buffer, pH 9.0, and resuspended in the same buffer. A freshly dissolved solution of fluorescein isothiocyanate (FITC) (3µg ml^{-1}) in 0.1 M-phosphate buffer, pH 9.0, was added dropwise to final concentration of 1 pµ ml^{-1}. FITC was allowed to bind covalently to protein during an 8 h storage in the refrigerator before unbound dye was washed off by centrifugation and the cells were resuspended in 0.02 M phosphate buffer, pH 7.4, containing 0.13 M NaCl. The emission was registered using the FITC (Boye et al., 1983).

References

Aqawi M, Sionov RV, Gallily R, Friedman M, Steinberg D (2021) Anti-bacterial properties of cannabigerol toward Streptococcus mutans. Front Microbiol 22:656471. https://doi.org/10.3389/fmicb.2021.656471

Boye E, Steen HB, Skarstad K (1983) Flow cytometry of bacteria: a promising tool in experimental and clinical microbiology. Microbiology. 1 :973-980. https://doi.org/10.1099/00221287-129-4-973

Steen HB, Jernaes MW, Skarstad K, Boye E (1994) Staining and measurement of DNA in bacteria. Methods Cell Biol. 477-487. Academic Press. https://doi.org/10.1016/S0091-679X(08)61091-2

Sugimoto A, Maeda A, Itto K, Arimoto H (2017) Deciphering the mode of action of cell wall-inhibiting antibiotics using metabolic labeling of growing peptidoglycan in Streptococcus pyogenes. Sci Rep 25:1-2. https://doi:10.1038/s41598-017-01267-5

Winson MK, Davey HM (2000) Flow cytometric analysis of microorganisms. Methods. 1:231-40. https://doi.org/10.1006/meth.2000.1003
